# Mineralocorticoid Receptor in Glutamatergic Neurons Modulates Anxiety Exclusively in Male Mice Via Regulation of the Actin-Bundling Factor Fam107a

**DOI:** 10.1016/j.bpsgos.2025.100651

**Published:** 2025-11-07

**Authors:** Huanqing Yang, Sowmya Narayan, Joeri Bordes, Lotte van Doeselaar, Carlo De Donno, Matthias Eder, Danusa Menegaz, Rosa-Eva Huettl, Lea-Maria Brix, Shiladitya Mitra, Margherita Springer, Marianne B. Müller, Alon Chen, Jan M. Deussing, Juan Pablo Lopez, Mathias V. Schmidt

**Affiliations:** a Research Group Neurobiology of Stress Resilience, Max Planck Institute of Psychiatry, Munich, Germany; b Research Group Translational Research in Psychiatry, Max Planck Institute of Psychiatry, Munich, Germany; c Institute of Computational Biology, Helmholtz Zentrum München, German Research Center for Environmental Health, Neuherberg, Germany; d Core Unit Electrophysiology, Max Planck Institute of Psychiatry, Munich, Germany; e Viral Vector Core Unit, Max Planck Institute of Psychiatry, Munich, Germany; f Leibniz Institute for Resilience Research, Mainz, Germany; g Translational Psychiatry, Department of Psychiatry and Psychotherapy, University Medical Center Mainz, Mainz, Germany; h Department of Molecular Neuroscience, Weizmann Institute of Science, Rehovot, Israel; i Research Group Molecular Neurogenetics, Max Planck Institute of Psychiatry, Munich, Germany; j Department of Neuroscience, Karolinska Institute, Stockholm, Sweden

**Keywords:** Anxiety, Behavior, Memory, Mineralocorticoid receptor, Sex, Stress

## Abstract

**Background:**

Exposure to stressful life events is a major risk factor for many psychiatric disorders. The mineralocorticoid receptor (MR) is a key regulator of the hypothalamic-pituitary-adrenal axis, a central stress response component. Stress-related mental disorders, such as anxiety and depression, are associated with MR dysfunction in the brain, but its cell type–specific contributions to emotional behavior and cognitive function remain unclear.

**Methods:**

Using a mouse model with a specific deletion of MR in forebrain glutamatergic neurons, we tested the behavioral, structural, and functional impact of MR in this neuronal population (*n* = 9–14 for behavioral and *n* = 3–4 for structural and functional analyses).

**Results:**

We revealed a specific function of MR in regulating baseline anxiety in male but not female mice. This distinct behavioral phenotype was associated with hippocampal structural and functional alterations. Furthermore, we identified a previously unrecognized downstream target of MR, the actin-bundling factor Fam107a, whose expression is tightly regulated by MR. Overexpression of Fam107a in the hippocampus was sufficient to rescue the increased anxiety phenotype of glutamatergic MR knockout mice.

**Conclusions:**

Together, our results underline the central role of MR as a potential target in understanding the intricate interplay between stress, resilience, and mental health.

## Plain Language Summary

Stressful experiences are a significant risk factor for psychiatric disorders, including anxiety and depression. At the core of the body’s stress response is the hypothalamic-pituitary-adrenal (HPA) axis, which is tightly regulated by the mineralocorticoid receptor (MR). While we know that MR dysfunction is linked to stress-related mental health issues, its specific function within different types of brain cells has been largely unknown. In this study, we investigated the role of MR in glutamatergic neurons, the brain’s primary excitatory cell type. Using a mouse model with a targeted deletion of the MR gene in these specific neurons, we discovered a key function of MR in regulating baseline anxiety. Notably, this effect was observed exclusively in male mice; female mice were unaffected. This sex-specific behavioral change was associated with structural and functional alterations in the hippocampus, a brain region critical for emotion and memory. Further investigation revealed a novel downstream target of MR: the actin-bundling protein FAM107a. We found that MR tightly regulates the expression of this protein. Remarkably, when we increased the expression of FAM107a in the hippocampus of the MR-deficient male mice, it was sufficient to partially rescue their increased anxiety phenotype. Our findings demonstrate a direct link between MR in glutamatergic neurons and anxiety, highlighting a previously unappreciated sex-specific mechanism. By identifying FAM107a as a critical downstream target, we have uncovered a new potential pathway for therapeutic intervention.

Mental health is crucial for navigating a rapidly changing world, influencing our ability to cope with stress, make decisions, and achieve goals. Poor mental health has a significant societal and economic impact, leading to decreased productivity, higher health care costs, and increased burden on families. The World Health Organization estimates that nearly 1 billion people have a psychiatric disorder (1,2). After the COVID-19 pandemic, the number of psychiatric patients increased dramatically, with the greatest increase in anxiety disorders and depression (3).

Psychiatric disorders are complex, caused by a mix of genetic and environmental factors. Stress is a major contributor, as intense or chronic stress can disrupt the body’s coping mechanisms, leading to conditions such as depression, anxiety, and posttraumatic stress disorder. The hypothalamic-pituitary-adrenal (HPA) axis is a key part of the stress response system, crucial for regulating how the body reacts to stressors (4). The mineralocorticoid receptor (MR) is an integral part of the HPA axis, and together with the glucocorticoid (GC) receptor (GR), it regulates its activity through negative feedback mechanisms (5). However, the cell type–specific contribution of the MR, especially to regulating emotional behavior, is not well understood.

As a nuclear receptor, MR is widely expressed in epithelial tissues, where it helps to maintain sodium and potassium homeostasis (6,7). MR is also found in the central nervous system, where it plays a significant role in endocrine control, emotional regulation, and cognitive function (8). Evidence increasingly suggests a link between MR and stress-induced mental disorders (9,10). It has been demonstrated in numerous clinical studies and animal models that MR dysfunction in brain regions connected to emotion and cognition can result in abnormal emotional behavior as well as learning and memory impairment (11, 12, 13). MR inhibition has repeatedly been associated with reduced anxiety in humans (14). Therefore, MR is emerging as a potential target for the complex interplay between stress, resilience, and mental health.

Because MR is expressed in various brain cell types, its contribution to endocrine and behavioral regulation is likely highly cell-type specific. Single-cell RNA sequencing data shows prominent MR expression in glutamatergic neurons (the brain’s main excitatory neuronal population), but the functional and behavioral role of MR in these neurons is not fully understood (15, 16). Mice with increased levels of MR in CaMKIIα (calcium/calmodulin-dependent protein kinase IIα)–expressing forebrain neurons, which include both glutamatergic and GABAergic (gamma-aminobutyric acidergic) subpopulations (17,18), exhibit decreased anxiety-like behavior in males (19,20), with less of an effect in females (21). The same mouse line was found to display decreased spatial memory and increased contextual fear memory (22). Consistent with these findings, overexpression of MR specifically in the basolateral amygdala reduced anxiety-related behavior and corticosterone (CORT) secretion in rats following an acute stressor (23). On the other hand, deletion of MR using the CaMKIIα promoter resulted in enhanced fear to cue during acquisition of the task, increased contextual fear memory, and impaired behavioral and endocrine adaptation (24). Similarly, pharmacologically blocking MR in the insula prevents the anxiety-reducing effects of CORT, suggesting that MR activation is crucial for GC-induced anxiolysis (25). However, mice with a forebrain and hippocampus deletion of MR driven by the Emx1 promoter were also reported to display reduced anxiety (26).

While the importance of MR in emotional and cognitive regulation is evident, the ambiguity surrounding MR’s cell type–specific function, particularly in glutamatergic neurons, limits understanding of MR function in relation to psychiatric and stress-related phenotypes. Therefore, here we undertook a series of studies to elucidate the molecular mechanisms and behavioral implications of MR function in this critical neuronal population.

## Methods and Materials

### Animals

The MR^lox/lox^-Nex-Cre mouse line was generated as previously described (27,28), with specific details regarding mouse breeding, genotyping primers, and standard housing conditions available in the Supplement.

### Stress Paradigms

Restraint stress was utilized for acute stress (29). Mice were restrained into 50 mL falcon tubes with a hole in the top and the lid, respectively. The acute restraint stress lasted for 30 minutes. At the end of 30 minutes, tail blood was collected from the mice (30), and blood samples were collected in 1.5 mL EDTA-coated microcentrifuge tubes (Kabe Labortechnik) for CORT level testing. The mice were then put back in their cages for 60 minutes, and tail blood was collected again at 90 minutes after the start of the experiment. The chronic social defeat stress (CSDS) paradigm is commonly used to induce anxiety- and depression-related behaviors in mice and was performed as previously described (31, 32, 33, 34). For details, see the Supplement.

### Behavioral Experiments

All behavioral tests were performed in a dedicated test room adjacent to the animal housing room. To prevent any potential behavioral alterations due to circadian rhythmic changes in CORT levels, tests took place between 8 am and 1 pm. The tests included the open field (OF), elevated plus maze (EPM), novel object recognition (NOR), spatial object recognition (SOR), and Morris water maze (MWM) tests. Among these, the OF and EPM tests are mostly used to assess the anxiety behavior of mice, while the NOR, SOR, and MWM tests are primarily used to assess the cognitive function of mice. The interval between behavioral tests was at least 24 hours to minimize carryover effects. The automated video tracking system ANY-maze (ANY-maze 6.18; Stoelting Co.) was used to record, track, and assess the tests. If a manual assessment was required, it was performed by skilled scientists who were blinded to the experimental conditions. For experimental details, see the Supplement.

### Electrophysiology

At the age of 8 to 12 weeks, mice were anesthetized with isoflurane and decapitated. The brain was rapidly removed from the cranial cavity, and using a vibratome (HM650V; Thermo Fisher Scientific), 350-μm-thick horizontal slices containing the ventral hippocampus were cut in an ice-cold carbogen gas (95% O_2_/5% CO_2_)–saturated solution consisting of (in mM) 87 NaCl, 2.5 KCl, 25 NaHCO_3_, 1.25 NaH_2_PO_4_, 0.5 CaCl_2_, 7 MgCl_2_, 10 glucose, and 75 sucrose. Slices were incubated in carbogenated physiological saline for 30 minutes at 34 °C and afterward for at least 30 minutes at room temperature (23–25 °C). The physiological saline contained (in mM) 125 NaCl, 2.5 KCl, 25 NaHCO_3_, 1.25 NaH_2_PO_4_, 2 CaCl_2_, 1 MgCl_2_, and 10 glucose. All measurements were conducted at room temperature. Details of long-term potentiation (LTP), paired-pulse facilitation (PPF), and patch-clamp experiments are described in the Supplement.

### In Situ Hybridization

In situ hybridization was performed as previously described (35). In short, brain slices at 20 μm were fixed in 4% paraformaldehyde, dehydrated, and exposed to a 35S-UTP-labeled antisense MR riboprobe. Following overnight hybridization at 55 °C, the sections were washed, dried, and exposed to Kodak Biomax MR films. Autoradiographs were digitized, and optical density for expression analysis was measured using ImageJ software (National Institute of Mental Health).

### RNAScope Analysis and Cell Counting

The RNAScope Fluorescent Multiplex Kit (320850; Advanced Cell Diagnostics) was used on 20-μm brain sections following manufacturer’s instructions. mm-Fam107a-C1, mm-Nr3c2-C2, and mm-scl17a6-C3 probes were used for staining. All images were acquired using a Zeiss laser scanning confocal microscope and Zen software, using a 40× objective (3 animals per marker and condition). All images were collected under the same imaging conditions. Each image was acquired in a 1.0-μm z-stack. The messenger RNA (mRNA) in the cells was quantified and analyzed using ImageJ and QuPath 0.3.2 software.

### Statistical Analysis

SPSS Statistics (version 22; IBM Corp.) software was used to analyze the data, and GraphPad Prism 8.0 was used to create the graphics (GraphPad Software). An independent Student’s *t* test was used to compare the 2 groups. The data were tested for normality using the Shapiro-Wilk test and quantile-quantile plot. If the data were not normally distributed, the nonparametric Mann-Whitney *U* test was applied. Data from more than 2 groups were analyzed with a 2-way analysis of variance (ANOVA) model followed by Tukey post hoc analysis to determine the statistical significance of any differences between groups. Time-series data were analyzed by a 2-way repeated-measures ANOVA followed by Tukey post hoc analysis. If behavioral data were not normally distributed, a log transformation was utilized before analysis. The ANOVA significance levels were set at *p* < .05 for main effects and *p* < .1 for interaction effects. The significance threshold was set at *p* < .05 for each post hoc test. Values outside 2 SDs were considered outliers and excluded from the analysis. Data are visualized as mean ± SEM.

## Results

### Validation of Cre-loxP-Dependent MR Knockout in Forebrain Glutamatergic Neurons and Its Effect on Anxiety-Related Behavior and Cognition at Baseline Levels

**Figure 1:**
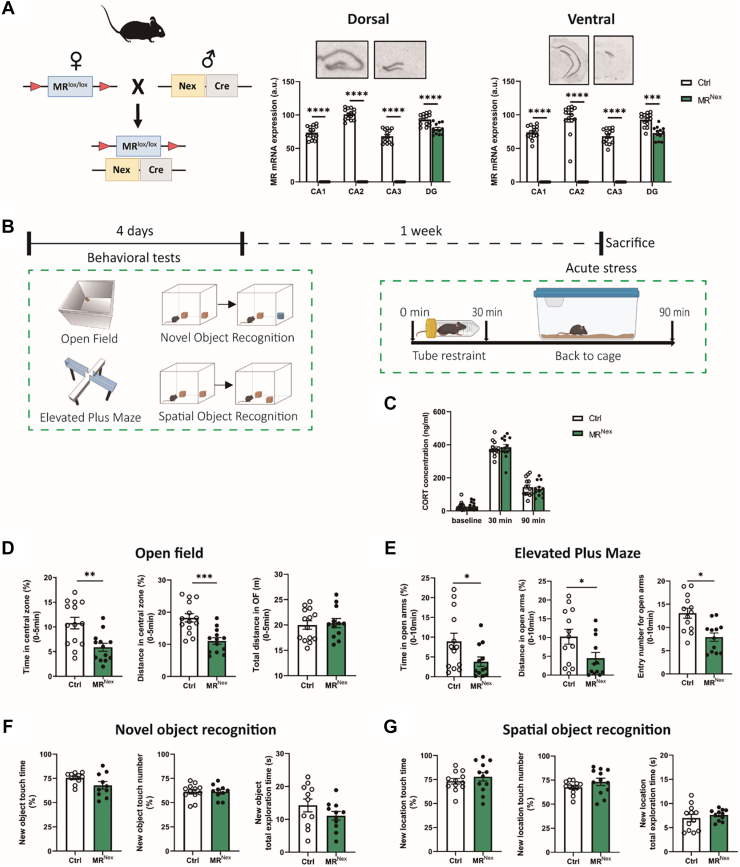
Male MR^Nex^ mice display increased anxiety. **(A)** Left panel: breeding scheme for MR^Nex^ mice. Right panels: MR mRNA expression levels in the dorsal and ventral hippocampus. **(B)** Experimental timeline. **(C)** CORT levels were significantly increased following acute restraint, with no genotype differences (time: *F*_2,73_ = 426.6, *p* < .0001; genotype: *F*_1,73_ = 0.009, *p* = .926). In the OF test **(D)** and EPM test **(E)**, lack of MR in glutamatergic neurons reveals an anxiety-like phenotype (OF: distance: *t*_25_ = −0.967, *p* = .343; time center: *t*_25_ = 3.396, *p* = .002; distance center: *t*_25_ = 4.325, *p* < .0001; EPM: open arm time: *t*_23_ = 2.107, *p* = .046; open arm distance: *t*_23_ = 2.2232, *p* = .036; open arm entries: *t*_23_ = 2.689, *p* = .013). The cognitive function of mice in the NOR test **(F)** or SOR test **(G)** was not significantly affected by the loss of MR in glutamatergic neurons. (NOR: duration novel object: *t*_19_ = 1.896, *p* = .073; frequency novel object: *t*_20_ = 0.001, *p* = .999; total object time: *t*_20_ = 1.327, *p* = .199; SOR: duration novel object: *t*_22_ = −0.856, *p* = .401; frequency novel object: *t*_22_ = −1.341, *p* = .193; total object time: *t*_21_ = −0.733, *p* = .472). Ctrl group: *n* = 14, MR^Nex^ group: *n* = 13. ∗Indicates a significant difference at ∗*p* < .05, ∗∗*p* < .01, ∗∗∗*p* < .001, ∗∗∗∗*p* < .0001. [Figure **(B)** created in BioRender.] a.u., arbitrary unit; CORT, corticosterone; Ctrl, control; DG, dentate gyrus; EPM, elevated plus maze; MR, mineralocorticoid receptor; mRNA, messenger RNA; NOR, novel object recognition; OE, overexpression; OF, open field; SOR, spatial object recognition.

We confirmed successful MR deletion in MR^Nex^ mice (Figure 1A). MR expression was undetectable in the CA1, CA2, and CA3 regions of MR^Nex^ mice. Next, we assessed baseline anxiety-related behavior and cognition as well as the endocrine stress response in MR^Nex^ mice (Figure 1B). CORT levels of both MR^Nex^ and control (Ctrl) mice increased following restraint (Figure 1C) and decreased after mice returned to their cages for an hour, independent of genotype.

Next, we tested the behavior of the animals. Even though there was no difference in the total distance traveled by MR^Nex^ and Ctrl mice in the entire OF arena, MR^Nex^ mice spent significantly reduced time and traveled significantly less distance in the central area of the OF compared with the Ctrl group (Figure 1D). The anxiogenic phenotype of MR^Nex^ mice was confirmed in the EPM. Comparing MR^Nex^ and Ctrl mice, the time spent in the open arms, the distance in the open arms, and the ratio entering the open arms were all significantly decreased (Figure 1E). On the cognitive level, neither the NOR test nor the SOR test revealed any statistically significant differences between MR^Nex^ and Ctrl mice in either the duration or frequency of touching novel objects or their total time spent exploring objects (Figure 1F, G). In a separate cohort of male mice, we assessed home cage locomotion and stressful learning in the MWM test (Figure S1A). For home cage locomotion, there were no discernible differences between Ctrl and MR^Nex^ mice (Figure S1B). For the MWM test, the average time it took for both genotypes to find the platform decreased during the 5 days of training, indicating that their capacity for learning was unhampered, with no genotype differences (Figure S1C, D).

Taken together, the data clearly indicate that mice lacking the MR in glutamatergic pyramidal neurons exhibit increased anxiety-like behavior, while their learning and memory ability is unaffected.

### Chronic Stress Exposure Has Mostly Genotype-Independent Effects in Male MR^Nex^ Mice

In addition to studying the phenotype of MR^Nex^ mice in basal conditions, we investigated whether emotional behavior and cognitive function are altered in this mouse line under chronic stress conditions. Therefore, mice underwent CSDS for 21 days, with behavioral testing on days 15 to 18 (Figure S2A). Basal CORT level at sacrifice showed a trend to be increased in the CSDS groups (Figure S2B), with no significant differences between genotypes.

We replicated the anxiogenic effect of glutamatergic MR deletion under nonstressed conditions in the OF, with a significant genotype effect for time and distance in the central area. CSDS also resulted in an increase in anxiety for both Ctrl and MR^Nex^ mice, independent of genotype. Also, the CSDS group moved in the entire box less than the nonstressed group overall (Figure S2C). In the EPM, the percentage of times the CSDS group entered the open arm and the time spent in the open arm were significantly reduced compared with the nonstressed group. MR^Nex^ mice spent less time and moved less distance compared with the Ctrl mice in the open arm, with no significant genotype × stress interaction (Figure S2D). In the NOR test, regardless of whether the mice received CSDS or not, there were no significant differences between MR^Nex^ and Ctrl mice. Furthermore, no significant interaction between genotype and stress was detected (Figure S2E). In the SOR test, we observed that while there was no obvious difference in the frequency of exploring the object in a new place or the total amount of time spent exploring objects, the percentage of MR^Nex^ mice touching the object in the new location was higher than that of Ctrl mice following CSDS (Figure S2F).

Taken together, the data demonstrate that mice exhibit increased anxiety in response to chronic stress, although CSDS mostly has genotype-independent effects in male MR^Nex^ mice compared with Ctrl mice.

### The Anxious Phenotype in MR^Nex^ Mice Is Sex Specific

Animals respond differently to stress depending on their sex. Previous research has demonstrated that female mice do not exhibit the same neuroendocrine alterations in response to persistent stress as male mice (33,36). Therefore, next we tested a separate cohort of female MR^Nex^ animals to assess any sex-specific behavioral effects. MR^Nex^ and Ctrl females were tested under nonstress conditions or exposed to the female 21-day CSDS paradigm, and behavioral testing was carried out on experimental days 15 to 18 (Figure S3A). Baseline CORT secretion was unaltered between genotypes and between stress treatments (Figure S3B). In the OF test (Figure S3C), there was no discernible difference between the MR^Nex^ and Ctrl mice whether they experienced CSDS or not. However, compared with nonstressed animals, animals that had experienced CSDS displayed increased anxiety, as indicated by decreased time spent in the central area of the OF arena and less movement in the whole OF arena. In the EPM test, there was no discernible difference between the MR^Nex^ and Ctrl mice whether they experienced CSDS or not. Similar to the OF results and consistent with previous reports (37), mice that experienced CSDS spent less time and moved less in the open arm (Figure S3D). No statistically significant differences were detected between stress conditions or genotypes in the NOR or SOR tests (Figure S3F).

Together, these data indicate that, in contrast to males, females do not display significant behavioral differences following MR deletion in glutamatergic forebrain neurons under basal or chronic stress conditions. Therefore, we continued to study the structural, functional, and molecular consequences of conditional MR deletion in male mice.

### Lacking MR in Forebrain Glutamatergic Neurons Alters Neurotransmission and Short-Term Plasticity at Hippocampal CA3-CA1 Synapses in Male Mice

**Figure 2:**
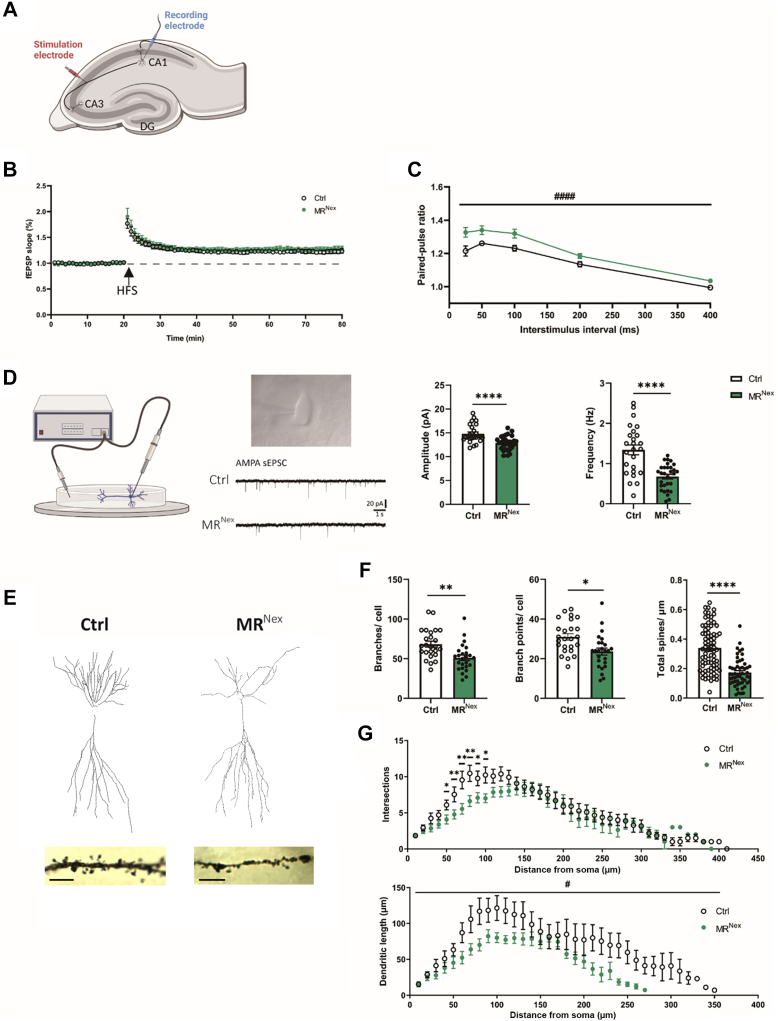
Deletion of MR in forebrain glutamatergic neurons leads to functional and structural alterations. **(A)** Schematic recording setup. **(B)** There is no significant difference between MR^Nex^ and Ctrl mice in long-term potentiation induction (*n* = 3 animals with 8–9 sections per group). **(C)** The paired-pulse ratio of MR^Nex^ mice was higher than that of Ctrl mice (*F*_1,90_ = 31.91, *p* = .0001; *n* = 3 animals with 10 sections per group). **(D)** Patch clamp recordings (left panel: schematic recording setup) revealed a lower amplitude (*t*_53_ = 4.221, *p* = .0001) and a reduced frequency (*t*_53_ = 5.194, *p* = .0001) of glutamate neurotransmitter release from presynaptic vesicles of MR^Nex^ mice. Wild-type: *n* = 4 animals, 25 sections; knockout: *n* = 4 animals, 30 sections. **(E)** Example pictures of neuronal morphology in MR^Nex^ and Ctrl mice. Scale bars: 5 μm. **(F)** MR deletion in glutamatergic neurons significantly reduced the dendritic branch number, branch points, and the number of spines in CA3 compared with Ctrl mice (dendritic length: *t*_48_ = 3.432, *p* = .001; dendritic branch points: *t*_48_ = 3.103, *p* = .003; spines: *t*_127_ = 7.036, *p* < .001). Data obtained from *n* = 3 mice per group. **(G)** Deleting MR in glutamatergic neurons significantly reduced the number of dendritic intersections and dendritic length. Sholl analysis revealed that MR loss had an effect on the complexity of dendritic branching, mainly at 50–100 μm away from the soma (50 μm: *p* = .041, 60 μm: *p* = .003, 70 μm: *p* = .002, 80 μm: *p* = .003, 90 μm: *p* = .011, 100 μm: *p* = .017). The deletion of MR in glutamatergic neurons also significantly reduced the dendritic length compared with the Ctrl group (*t*_60_ = 2.174, *p* = .034). Data obtained from *n* = 4 mice per group. ^#^Indicates genotype effect with ^#^*p* < .05, ^####^*p* < .0001. ∗Indicates significant group difference with ∗*p* < .05, ∗∗*p* < .01, ∗∗∗∗*p* < .0001. [Figure **(A, D)** created in BioRender.] Ctrl, control; DG, dentate gyrus; fEPSC, field excitatory postsynaptic potential; HFS, high frequency stimulation; MR, mineralocorticoid receptor; sEPSC, spontaneous excitatory postsynaptic potential.

Next, we investigated whether MR deletion affects synaptic transmission and plasticity in the ventral CA3-CA1 network. First, we performed field potential recordings at CA3-CA1 synapses in acute brain slices to test for changes in LTP and PPF (Figure 2A). The magnitude of LTP did not differ between MR^Nex^ and Ctrl mice (Figure 2B), which is consistent with the unaltered cognitive abilities of the mice. However, PPF, a presynaptic form of short-term synaptic plasticity, was more pronounced in slices from MR^Nex^ mice (Figure 2C), suggestive of a lower neurotransmitter release probability at CA3-CA1 synapses in MR^Nex^ mice. To corroborate this finding, we performed patch-clamp recordings of AMPA receptor–mediated miniature excitatory postsynaptic currents (AMPA-mEPSCs) in CA1 pyramidal cells, expecting a lower mEPSC frequency for the MR^Nex^ condition. In slices from MR^Nex^ mice, both the amplitude and frequency of AMPA-mEPSCs was decreased compared with recordings in slices from Ctrl animals (Figure 2D). Taken together, these data show that MR inactivation in forebrain glutamatergic neurons has a significant impact on glutamatergic signaling in the ventral hippocampus.

### Deletion of MR in Glutamatergic Pyramidal Neurons Leads to Structural Changes in the Male Hippocampus

Next, we assessed whether deleting MR in glutamatergic pyramidal neurons has an effect on neuronal morphology (Figure 2E). As shown in Figure 2F, deletion of MR in glutamatergic neurons significantly reduced the dendritic length as well as the number of spines in CA3 compared with Ctrl mice (Figure 2F and Figure S4). Sholl analysis of the number of dendritic interactions further revealed that MR loss had an effect on the complexity of dendritic branching, mainly 50–100 μm away from the soma (Figure 2G). This suggests that MR deletion mainly affects the midsegment of dendritic branches. Moreover, the deletion of MR in glutamatergic neurons significantly reduced the dendritic length compared with the Ctrl group (Figure 2G).

### Single-Cell RNA Sequencing Identifies Fam107a as an MR Target Gene

**Figure 3:**
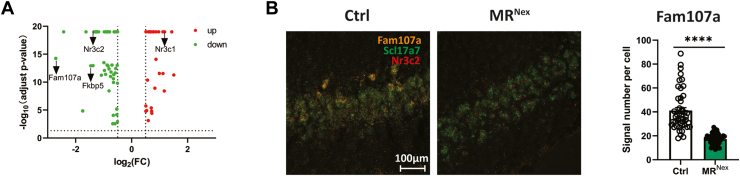
Single-cell RNA sequencing data of MR^Nex^ animals. **(A)** Volcano plot showing the log_2_FC pattern of differential genes. **(B)** Validation of Fam107a downregulation in MR^Nex^ mice (*t*_92_ = 9.274, *p* < .0001). ∗Significant group difference at ∗∗∗∗*p* < .0001. Ctrl, control; FC, fold change; MR, mineralocorticoid receptor.

In order to investigate downstream mediators that could be responsible for the structural, functional, and behavioral alterations observed after MR deletion in glutamatergic neurons, we screened for differentially expressed genes (DEGs), using a single-cell RNA sequencing dataset from the ventral hippocampus of wild-type and MR^Nex^ mice exposed to acute stress, as well as nonstressed Ctrl mice (16). Interestingly, a subcluster of CA3 neurons (CA3 Glut2) showed the largest number of DEGs between Ctrl and MR^Nex^ mice under basal conditions. The same CA3 Glut2 cluster was also overrepresented after acute stress exposure. Therefore, we further focused our analysis on this neuronal subtype (Figure S5A). More specifically, we focused on DEGs identified both at baseline (MR^Nex^ vs. wild-type) and after acute stress exposure (stress vs. Ctrl), with a log_2_ fold change >2 (up or down), which yielded a list of 78 DEGs within the CA3 Glut 2 cluster (Figure 3A). Among these genes, we confirmed regulation of *Nr3c2* (MR) and known target genes (*Nr3c1* [GR], *Fkbp5*) (38). Intriguingly, we also identified *Fam107a* as one of the most strongly downregulated genes in the CA3 Glut2 cluster in MR^Nex^ mice. We and others had previously shown that *Fam107a*, also known as DRR1, is a stress-responsive actin-bundling factor that is involved in synaptic plasticity and stress resilience (39,40). The robust downregulation of Fam107a in MR^Nex^ mice was confirmed in an independent set of animals using RNAScope (Figure 3B). Therefore, we selected this gene for further functional analyses.

### Overexpression of Fam107a Partially Rescues the Anxiety Phenotype of MR^Nex^ Mice

**Figure 4:**
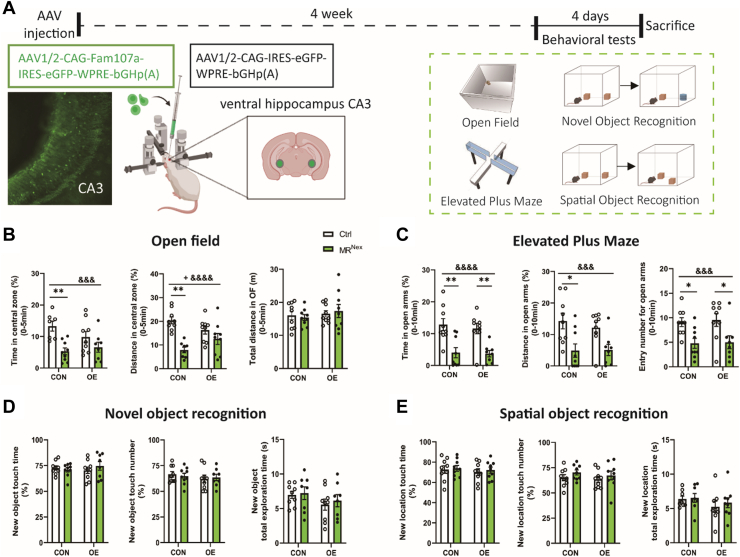
CA3-specific overexpression partially rescues the anxiogenic phenotype of male MR^Nex^ mice. **(A)** Experimental design. **(B)** In the OF, control virus–injected MR^Nex^ mice spent significantly less time and covered significantly less distance in the central zone. This effect was absent in Fam107a-OE mice (*F*_1,31_ = 6.466, *p* = .016). **(C)** In the elevated plus maze, MR^Nex^ mice displayed reduced open arm entries and time depending of Fam107a expression (open arm duration: *F*_1,31_ = 27.53, *p* < .0001; open arm distance: *F*_1,31_ = 15.99, *p* < .0001; open arm entries: *F*_1,32_ = 15.15, *p* < .0001). In the NOR test **(D)** and SOR test **(E)**, no significant genotype or Fam107a effects were detected. NOR: overexpression effects: percentage of time to explore the new object: *F*_1,31_ = 0.076, *p* = .784; frequency of exploring new object: *F*_1,31_ = 0.883, *p* = .355; total time spent exploring new object: *F*_1,30_ = 2.530, *p* = .122; genotype effects: percentage of time to explore the new object: *F*_1,31_ = 0.181, *p* = .673; frequency of exploring the new object: *F*_1,31_ = 0.005, *p* = .944; total time spent exploring the new object: *F*_1,30_ = 0.271, *p* = .606. SOR: overexpression effects: percentage of time to explore the new position: *F*_1,32_ = 0.476, *p* = .495; frequency of exploring the new position: *F*_1,32_ = 0.488, *p* = .489; total time spent exploring the new position: *F*_1,29_ = 1.831, *p* = .186; genotype effects: percentage of time to explore the new position: *F*_1,__32_ = 0.246, *p* = .623; frequency of exploring the new position: *F*_1,32_ = 1.915, *p* = .176; total time spent exploring the new position: *F*_1,29_ = 0.325, *p* = .573. Ctrl CON: *n* = 9, Ctrl OE: *n* = 9, MR^Nex^ CON: *n* = 9, MR^Nex^ OE: *n* = 9. ^&^Indicates a virus effect at ^&&&^*p* < .001, ^&&&&^*p* < .01. ^+^Indicates a genotype × virus interaction effect at ^+^*p* < .05. ∗Indicates a post hoc test at ∗*p* < .05, ∗∗*p* < .01. [Figure **(A)** created in BioRender.] AAV, adeno-associated virus; CON, control virus–injected; MR, mineralocorticoid receptor; NOR, novel object recognition; OE, overexpressing; OF, open field; SOR, spatial object recognition.

To test our hypothesis that the MR-mediated downregulation of *Fam107a* contributes to the male-specific increase in anxiety-related behavior, MR^Nex^ and Ctrl mice were injected in the CA3 region of the hippocampus with either a *Fam107a*-expressing virus AAV1/2-CAG-FAM107A-IRES-eGFP-WPRE-bGHp(A) or a Ctrl virus AAV1/2-CAG-IRES-eGFP-WPRE-bGHp(A) (Figure 4A). Successful viral expression was confirmed via immunofluorescence (Figure 4A). Then, we performed behavioral tests on the mice 4 weeks after virus injection. In the OF test (Figure 4B), we found a significant interaction effect between genotype and overexpression of Fam107a on anxiety-like behavior. The anxiogenic phenotype induced by MR deletion in glutamatergic neurons was ameliorated under Fam107a overexpression conditions; however, this comparison did not reach statistical significance in the post hoc comparison. In contrast, for the EPM, the main genotype effect was not modulated by Fam107a overexpression (Figure 4C). We also checked for cognitive effects in the NOR (Figure 4D) and SOR (Figure 4E) tests but did not find significant effects.

### Overexpression of Fam107a in CA3 Glutamatergic Neurons Partially Reverses the Anxiety-Related Phenotype of MR^Nex^ Mice

The moderate effect observed following Fam107a overexpression could be due to the lack of cell-type specificity. To establish a specific role of Fam107a in glutamatergic neurons, we next designed a Cre-dependent Fam107a overexpression virus to specifically target the CA3 glutamatergic cell population. First, we tested the influence of this cell type–specific Fam107a overexpression compared with a Ctrl virus injection under wild-type conditions using Nex-Cre mice (Figure S6A). The successful and cell type–specific overexpression of Fam107a in the CA3 area of the hippocampus was confirmed by RNAScope (Figure S6B). Interestingly, we observed that Fam107a overexpression in glutamatergic neurons was sufficient to reduce anxiety-like behavior in the OF (Figure S6C). While there was no significant change in the overall distance traveled, the Fam107a-overexpressing animals spent significantly more time and moved a greater distance in the central part of the OF. Similar to the effects observed by the cell type–nonspecific Fam107a overexpression, the anxiolytic effect was limited to the OF and was not observed in the EPM (Figure S6D). Here, we did not observe any significant differences. Furthermore, no effects were observed for cognitive behavior in the NOR and SOR tests (Figure S6E, F).

**Figure 5:**
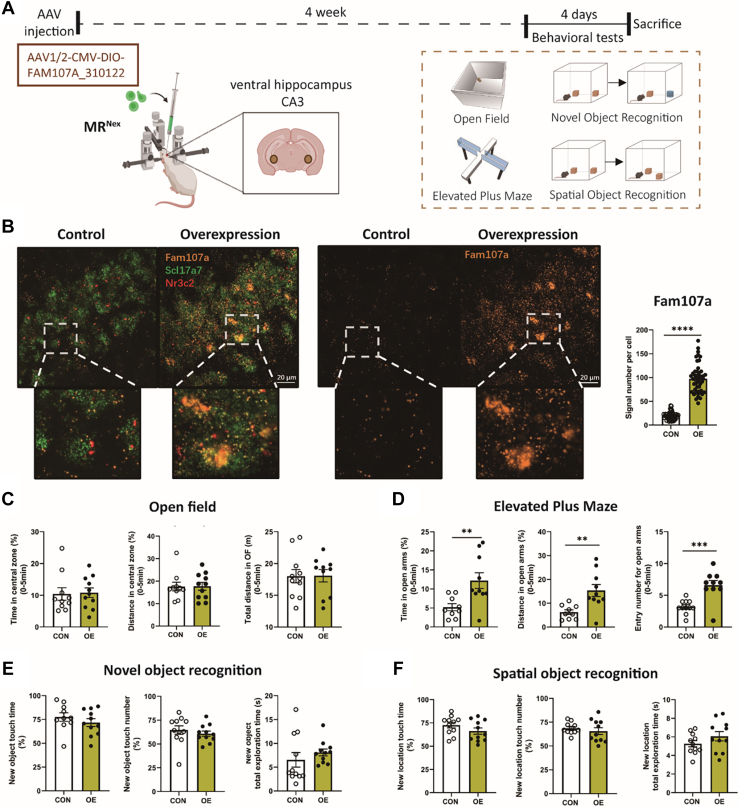
Injection of Cre-dependent Fam107a DIO-AAV virus on MR^Nex^ mice can partially rescue the genotype. **(A)** For Fam107a overexpression, Cre-dependent DIO-AAV virus was injected into both sides of the ventral hippocampus CA3 region in mice. **(B)** The expression of Fam107a mRNA in glutamatergic CA3 neurons was significantly increased in Fam107a OE mice compared with control virus–injected mice (*t*_98_ = 18.01, *p* < .0001). Left panel: RNAscope images showing the mRNA expression of Fam107a (orange), MR (Nr3c2; red), and glutamatergic neuron-specific markers (Scl17a7; green). Right panel: individual channel RNAscope images showing the mRNA expression of Fam107a (orange). Pictures are taken from the ventral CA3 area as indicated in the brain map in **(A)**. **(C)** In the OF test, no significant effects of Fam107a overexpression were detected (time spent in the central zone: *t*_19_ = 0.156, *p* = .877; central zone distance: *t*_19_ = 0.032, *p* = .975; total distance: *t*_19_ = 0.046, *p* = .964). **(D)** In the elevated plus maze, MR^Nex^ animals that overexpressed Fam107a spent significantly more time, covered more distance, and had more entries in the open arms than controls. Fam107a overexpression exclusively in hippocampal glutamatergic neurons led to significantly increased frequency of entering the open arm (*t*_17_ = 4.401, *p* < .0001), the open arm duration (*t*_17_ = 2.979, *p* = .008), and distance in the open arms (*t*_17_ = 3.232, *p* = .005). In the NOR **(E)** and SOR **(F)** tests, no significant differences were detected. NOR: percentage of time to explore the new object (*t*_19_ = 0.982, *p* = .338), frequency of exploring new object (*t*_20_ = 0.721, *p* = .479), total time spent exploring new object (*t*_20_ = 0.921, *p* = .368). SOR: percentage of time to explore the new position (*t*_20_ = 1.400, *p* = .177), frequency of exploring the new position (*t*_20_ = 0.741, *p* = .467), and total time spent exploring the new position (*t*_20_ = 1.334, *p* = .197). CON: *n* = 11, OE: *n* = 11. ∗Indicates a significant difference with ∗∗*p* < .01, ∗∗∗*p* < .001. [Figure **(A)** created in BioRender.] AAV, adeno-associated virus; CON, control virus–injected; MR, mineralocorticoid receptor; NOR, novel object recognition; OE, overexpressing; OF, open field; SOR, spatial object recognition.

Next, we tested whether Fam107a overexpression in glutamatergic CA3 neurons can reverse the anxiolytic phenotype of male MR^Nex^ mice (Figure 5A). The successful and cell type–specific overexpression of Fam107a in the CA3 area of the hippocampus was confirmed by RNAScope (Figure 5B). In the OF (Figure 5C), overexpression of Fam107a did not significantly alter the time spent in the central zone, the central zone distance, or the total distance. In contrast, in the EPM (Figure 5D), MR^Nex^ animals overexpressing Fam107a were significantly less anxious compared with animals injected with a Ctrl virus. Fam107a overexpression exclusively in hippocampal glutamatergic neurons led to significantly increased frequency of entering the open arm, open arm duration, and distance in the open arms. Cognition was again unaffected, with no significant difference on the NOR test (Figure 5E) or SOR test (Figure 5F).

## Discussion

MR is a critical factor regulating the stress response that has been implicated in the causal mechanisms underlying stress-related psychiatric disorders (13,41). Similarly, glutamatergic neuronal signaling is involved in a wide range of physiological processes, including the regulation of emotions (42,43), and consequently plays a crucial role in the pathophysiology of psychiatric disorders (44, 45, 46, 47, 48, 49). Here, we show that MR in glutamatergic neurons is specifically important in male mice to regulate baseline anxiety, while MR-mediated effects on cognition and HPA axis regulation are not affected by this neuronal population. Furthermore, with *Fam107a* we identify a previously unrecognized MR target gene, which as a stress-regulated actin-bundling factor can mediate the MR-dependent effects on anxiety-related behavior.

In the brain, MR is strongly expressed in neuronal cell types, most prominently excitatory glutamatergic and inhibitory GABAergic neurons (15). However, previous genetic and pharmacological studies have already implicated the MR in the regulation of emotional behavior with some contradicting results. While deletion of MR in CA2 or hippocampal MR antagonist treatment reduced anxiety (50, 51, 52), deletion of MR in all neuronal populations or forebrain CaMKII-expressing neurons had no effect on anxiety (27,52). The current data establish that the regulation of anxiety via MR seems to be specific to the glutamatergic neuronal population. While no effects on spatial cognition were detected, we cannot exclude effects of MR deletion on other forms of cognition, including habit learning (53). Furthermore, the lack of effect of chronic stress on performance in the spatial memory tests may indicate that these assays were not sensitive enough to detect subtle cognitive deficits. It is plausible that the task’s relatively low difficulty level masked potential impairments that might have been revealed by a more challenging cognitive assessment. Deletion of MR in glutamatergic neurons also altered neuronal structure and affected glutamatergic transmission and short-term synaptic plasticity in the hippocampus, which likely contributed to the altered behavioral phenotype. MR was previously described to be involved in the regulation of glutamatergic transmission, ensuring its proper functioning (54), predominantly through nongenomic effects (55, 56, 57). Similarly, CORT was shown to modulate spine morphology mediated via MR (58). Our data support these findings and indicate that the absence of MR in glutamatergic neurons leads to a presynaptic deficit in glutamate release, evidenced by enhanced PPF and a lower mEPSC frequency, which likely underlies the observed lower neuronal complexity, especially in the CA3 region of the hippocampus. Interestingly, despite this presynaptic deficit, the overall capacity for synaptic plasticity, as measured by LTP, remains intact, suggesting a dissociation between basal release properties and the LTP of synapses. This combination of effects likely contributes to the increased anxiety phenotype in our MR^Nex^ knockout (KO) mice. However, as the Nex-Cre deletes MR in all forebrain glutamatergic neurons, additional contributions of other brain regions like the prefrontal cortex cannot be ruled out. Furthermore, previous studies pointed to a compensatory change in GR expression following MR manipulation (26,38), which could also contribute to the phenotype.

Another important observation is that the anxiety-regulating role of MR appears to be sex specific. While the increased anxiety of male mice lacking MR in forebrain glutamatergic neurons was consistently observed in different behavioral tests and across several cohorts, the anxiety levels of female mice were not affected. These results imply that anxiety regulation in both sexes differs substantially also on a molecular level, further substantiating previous reports (59,60). Interestingly, chronic stress increased anxiety in both males and females, independent of glutamatergic MR levels, indicating that the anxiolytic effects of chronic stress exposure are not dependent on glutamatergic MR signaling.

Our study also identified *Fam107a* as a prominent and previously undiscovered MR target gene, whose expression showed a significant decrease in the hippocampus of mice lacking MR in glutamatergic neurons. The Fam107a gene encodes the DRR1 protein binding to F-actin (40). Fam107a was found to be predominantly expressed in the brain, especially in limbic system neurons (61,62). Initially identified as a downregulated gene in renal cell carcinoma, subsequent studies found it to be involved in a variety of physiological processes, including cell proliferation, differentiation, apoptosis, and stress response (63). In multiple studies, it has been discovered that Fam107a has differential expression in psychiatric disorders such as schizophrenia, bipolar disorder, and Alzheimer’s disease (64, 65, 66), suggesting its involvement in the pathophysiology of these disorders. It is interesting to note that previous coexpression and coregulation studies showed that Fam107A and AMPA receptors share similar promoter profiles and that they are coregulated and coexpressed (67). Accordingly, Fam107A could be the missing link between modulation of MR activity in glutamatergic neurons and changes in their electrophysiological properties through AMPA receptor regulation. It has also been reported that the protein expression of Fam107a in the brain is affected by GCs (68), and various stressors significantly increase Fam107a mRNA expression, especially in the paraventricular nucleus of the hypothalamus and the CA3 region of the hippocampus (40,65). Therefore, Fam107a is involved in stress-induced in vivo changes and plays a role in buffering the consequences of stress, potentially restoring brain homeostasis. Here, we demonstrated that Fam107a is associated with the anxiety-like phenotype in mice lacking MR in glutamatergic neurons. Increase of Fam107a expression either in all CA3 neurons or specifically in glutamatergic CA3 neurons was sufficient to partially rescue the increased anxiety phenotype of glutamatergic MR KO mice.

This study has a few limitations. Our study primarily examined Fam107a at the mRNA level due to a lack of specific antibodies. We also did not include structural or functional analyses in females, although no behavioral phenotype was observed in female mice. Lastly, we did not perform structural or functional analyses after Fam107a overexpression, which would have further supported the link to observed behavioral changes.

### Conclusions

We have shown that MR signaling in male mice’s glutamatergic neurons is essential for maintaining normal anxiety-related behavior. This effect is partly mediated by Fam107a, a stress-regulated actin-bundling factor and newly identified MR target, that influences structural, functional, and behavioral outcomes. These findings have significant implications for understanding and treating stress-related mental disorders.

## Supplementary Material

Supplemental Methods and Figures S1–S6

Key Resources Table
