# Supplementary material for: Mineralocorticoid Receptor in Glutamatergic Neurons Modulates Anxiety Exclusively in Male Mice Via Regulation of the Actin-Bundling Factor Fam107a
Source: Biol Psychiatry Glob Open Sci. 2025 Nov 7;6(2):100651. doi: 10.1016/j.bpsgos.2025.100651 (PMC12803904; doi:10.1016/j.bpsgos.2025.100651)
Supplement: Supplemental Methods and Figures S1–S6 [file mmc1.pdf]

SUPPLEMENTARY INFORMATION

Mineralocorticoid Receptor in Glutamatergic Neurons Modulates Anxiety Exclusively in Male Mice via Regulation of the Actin Bundling Factor FAM107a

Yang *et al.*

Supplemental Methods

Generation of $MR^{lox/lox}$-Nex-Cre mouse line

The creation of MR-floxed mice was previously described (1). To conditionally knock out MR in forebrain glutamatergic neurons, $MR^{lox/lox}$ mice, where exon 3 is flanked by loxP sites, were crossed with Nex-Cre animals (2). The transcription factor with a helix-loop-helix structure Nex (also known as NeuroD6/Math2) is a marker of neuronal precursors in the embryonic cortex. It is active in glutamatergic neurons in the neocortex, hippocampus, amygdala, and olfactory bulb of the adult mouse brain, and it is highly expressed in dorsal differentiated neurons of the telencephalon. Cre activity begins during development, starting from embryonic day 11.5. The resulting offspring with a deletion of MR in glutamatergic forebrain neurons ($MR^{lox/lox}$-Nex-Cre, referred to as MR^{Nex}) and their control $MR^{lox/lox}$ littermates (referred to as Ctrl) were used. All mouse lines used were on a mixed 129S2/Sv×C57BL/6 genetic background backcrossed to C57BL/6NRj. All animals used in single experiments were littermates. Experimenters were always blind to genotype.

Genotyping

Genotyping was performed by PCR using the following primers: MR-flox-7, 5´- CTG-GAG-ATC-TGA-ACT-CCA-GGC-T-3´; MR-flox-10, 5´-TAG-AAA-CAC-TTC-GTA-AAG-TAG-AGC-T-3´ and MR-flox-8, 5´-CCT-AGA-GTT-CCT-GAG-CTG-CTG-A-3´. Standard PCR conditions resulted in a 285-bp wild-type and a 335-bp floxed PCR product. In Nex-Cre mice, the presence of Cre was evaluated using the primers CRE-F 5′-GAT-CGC-TGC-CAG-GAT-ATA-CG-3′, CRE-R 5′-AAT-CGC-CATCTT- CCA-GCA-G-3′, Thy-F 5´-TCT-GAG-TGG-CAA-AGG-ACC-TTA-GG-3´ and Thy-R 5´-CCA-CTG-GTG-AGG-TTG-AGG-3´, resulting in a Cre-specific PCR product of 574 bp and a control PCR product of Thy1 of 372 bp.

Standard housing conditions

All studies were conducted in conformity with the European Communities' Council Directive 2010/63/EU, as well as the Guidelines for the Care and Use of Laboratory Animals of the Government of Upper Bavaria, Germany. The mice used in the experiments were all ranged in age from 8 to 20 weeks and both sexes were included in separate cohorts. Unless otherwise specified, during the experiment mice were housed individually in cages (IVC; 30 cm × 16 cm × 16 cm; 501 cm^2) with adequate bedding and nesting material and wooden tunnels for environmental enrichment 1 week prior to behavioral testing or hormonal assessment. Animals

were kept in a standard laboratory environment with *ad libitum* food (Altromin 1318, Altromin GmbH, Germany) and water, a central airflow system (Tecniplast, IVC Green Line-GM500), and maintained in a 12:12 hours light-dark cycle (lights on at 07.00 a.m.) at a constant temperature of 23±2 °C and 55% humidity. Semi-randomly chosen experimental groups were assigned to the animals, and data analysis was performed blinded to group assignment.

Chronic social defeat stress paradigm

For males, mice in the CSDS group were directly placed in the home cage of a new dominant CD1 male every day. The animals were separated by a perforated divider following an aggressive fight and 2-3 attacks or after a maximum of 5 minutes, thereby avoiding any injuries on the test animals. For female mice, in order to provoke a male CD1 resident attack, they were first covered with urine, which was collected from C57Bl/6n male mice previously and kept at room temperature (3). A brush was used to apply the urine to several parts of the body (head, back, tail and especially at the vaginal orifice. During the defeat, if male CD1 mice exhibited mating-like behavior, they were immediately separated. Defeat times were randomly assigned between 1 p.m. and 4 p.m. in the afternoon. On each day after the defeat, the mice in the CSDS group spent the following 24 hours in the same cage with the CD1 mice that attacked them, separated by a transparent and perforated panel. Animals in the control group were kept in their home cages for 21 days under general animal facility husbandry. During the experiment, all mice were weighed every week, and the fur condition was scored daily (4).

Assessment of home cage behavior

Home cage locomotion test was conducted on animals in order to evaluate their basal activity. The mice were individually placed in cages equipped with an infrared mouse motion detector/data logger (Mouse-E-Motion; Infra-E-Motion, Hamburg, Germany). Measurements were performed for 72 consecutive hours, during which time mice had free access to food and water. The data are displayed as total number of seconds in which any movement occurred in 30-minute intervals.

Open Field Test

The OF test is commonly utilized in studies of exploratory behavior and anxiety-like behaviors. As previously described (5,6), the open field used for the test is made of gray polyvinyl chloride plastic material with a size of 50 cm x 50 cm x 50 cm. The test lasted for a total of 15 minutes in low light conditions (about 15 lux). During the test, the mice were placed in a corner of the

open field and were allowed to explore the arena freely. The results were evaluated by dividing the total period into three 5-min segments, to analyze the distance the mice moved in the central area, the corners, and the entire open field, and time the mice spent in those areas.

Elevated Pluse Maze Test

The EPM test is used to assess anxiety-like behavior. As previously described (5,6), the elevated maze apparatus used in the test was made of grey polyvinyl chloride plastic material with two opposing open arms (30 cm × 5 cm × 0.5 cm) and two opposing closed arms (30 cm × 5 cm × 15 cm), connected into a cross shape by a middle platform (5 cm × 5 cm) with a height of 50 cm. The test was conducted under low light settings (approximately 10 lux in the closed arms and 20 lux ins the open arms) for a total of 10 minutes. During the test, the mice were placed on the middle platform area facing the closed arm, and allowed to explore the arms freely. The results were evaluated analyzing the percentage of the distance the mice moved in the open arms, the percentage of time spent in the open arms, and the number of times the mice entered the open arms.

Novel Object Recognition and Spatial Object Recognition Tests

Compared with familiar objects, rodents naturally tend to spend more time exploring unfamiliar one (7). The NOR test assess recognition memory and learning, while the SOR test assesses spatial memory. The objects used in the experiment were made by 13 Lego® bricks. For the NOR, two different objects variable in shape and color yet created to attain a consistent volume were used, while for the SOR two identical objects were used. To eliminate the mice's unfamiliarity, the experimental arena uses a large open home cage (425 x 276 x 153 mm), with bedding on the bottom of the cage. At the beginning of the experiment, the mice were given a 15-minute familiarization period, and two same objects were placed 5 cm from the rear of the arena, and the mice were then placed in the arena away from the objects and allowed to explore freely. Following the familiarization period, the mice were returned to their home cages for 20 minutes before a 5-minute testing period. In the NOR, one of the two identical objects was replaced by a different object in the arena, the mouse was placed in the same position of the arena as before. For the SOR, one of the objects was moved to a novel location, while the other one remained in the same location. Once the mouse touched an object with its nose, front paws, or whiskers, it was considered an interaction. Videos were manually scored using ANY-maze software, analyzing the ratio of time, and number of times the mice explored objects.

Morris Water Maze Test

The MWM test is a cognitive task designed to evaluate spatial learning and memory in mice. In our experiment, the MWM test was carried out in a white circular pool with a diameter of 150 cm and a depth of 41 cm. The pool (W 309 cm × L 357 cm × H 283 cm) was placed in the center of the room with visual cues in the room on the surrounding walls. The pool was filled to a depth of 33 cm, and the water temperature was maintained at 22-23°C throughout the test. The room is illuminated with indirect light and the light on the water surface reaches 11.5 lux. The escape platform is a cylinder made of 8 cm × 8 cm transparent acrylic plexiglass, which is placed in the center of the north-east (NE) quadrant, 35 cm away from the wall, and submerged 1 cm below the water surface.

First, the mice were allowed to adapt to the pool and water for one day, after which they underwent a 5-day training period to find the platform. Each animal performed 4 trials per day. The starting points for each of the four trials were randomly distributed along the south-west (SW) area of the tank's perimeter and were each used for a different starting position. The mouse was gently lowered into the water facing the wall and was allowed to explore the pool for a maximum of 90 seconds. If the platform was located, it was allowed to stay on the platform for 10 seconds. If the mouse did not find the platform within 90 seconds, it was guided onto the platform without touching it and remained there for 10 seconds before being taken from the pool. Inter-trial intervals were 15 minutes, which the mice spent in their home cage under a heating lamp to avoid hypothermia. On day 6, mice were subjected to a 60 seconds probe trial, for which the platform was removed. Time to reach the platform and time and distance travelled in each of the quadrants was recorded.

Electrophysiology

For long-term potentiation (LTP) and paired-pulse facilitation (PPF) experiments, slices were superfused with carbogenated physiological saline (4-5 ml/min flow rate). Field excitatory postsynaptic potentials (fEPSPs) at CA3-CA1 synapses were evoked by square-pulse electrical stimuli (50 µs pulse width) delivered via a bipolar tungsten electrode (50 µm pole diameter, ~0.5 MΩ nominal impedance) to the Schaffer collateral-commissural pathway. fEPSPs were recorded using glass microelectrodes (filled with physiological saline, ~1 MΩ open-tip resistance) that were placed into the CA1 stratum radiatum. The intensity of voltage stimulation was adjusted in a manner to produce a fEPSP of ~50% of the amplitude at which a population spike appeared. Recording data were low-pass filtered at 1 kHz and digitized at 5 kHz. Before

and after LTP induction, a single stimulation pulse was delivered every 15 s to the neural tissue. LTP was induced by high-frequency stimulation (HFS, 100 Hz for 1 s). Paired-pulse ratio was calculated by dividing the slope of fEPSP2 by the slope of fEPSP1.

For patch-clamp experiments, infrared videomicroscopy was used to identify individual CA1 pyramidal neurons. Somatic whole-cell voltage-clamp recordings from these cells (-70 mV holding potential, >1 GΩ seal resistance, <20 MΩ series resistance, 10 mV liquid junction potential correction, 3 kHz low-pass filter, 15 kHz sampling rate) were performed with an EPC 10 amplifier (HEKA). The carbogenated physiological saline (2-3 ml/min flow rate) additionally contained APV (50 µM), picrotoxin (100 µM), and TTX (1 µM). Patch pipettes (3-5 MΩ open tip resistance) were filled with a solution consisting of (in mM): 125 $CsCH_3SO_3$, 8 NaCl, 10 HEPES, 0.5 EGTA, 4 Mg-ATP, 0.3 Na-GTP, and 20 Na_2-Phosphocreatine. 10 min after break-in to the cell, AMPA receptor-mediated miniature excitatory postsynaptic currents (AMPA-mEPSCs) were recorded for 5 min. Offline analysis was performed using the Mini Analysis Program (Synaptosoft).

Corticosterone measurements

All blood samples were collected and stored on ice, then centrifuged at 8000 rpm for 15 minutes at 4°C. Afterwards, 10 µl of plasma was transferred to newly labelled microcentrifuge tubes and stored in a -20°C freezer. CORT levels were assessed by Double Antibody 125I Radioimmunoassay Kit (MP Biomedicals Inc., Eschwege, Germany; sensitivity 12.5 ng/ml) or Enzyme-linked Immunosorbent Assay (ELISA) kit (RE52211, TECAN, IBL Hamburg, Germany) following the manufacturer's instructions.

Immunohistochemistry

The perfused and fixed brains were removed and 40 µm thick sections were prepared using a vibrating microtome (Microm HM 650 V, Thermo Fisher Scientific). After blocking, the sections were incubated with primary antibody (Rabbit anti-Fam107a (ab185459), Abcam, 1:1000) at 4°C for overnight and protected from light. The next day, sections are washed and incubated with the appropriate fluorescence-conjugated secondary antibody (Goat anti-Rabbit IgG Alexa Fluor 488; Invitrogen, Thermo Fisher Scientific, 1:500) for 2 hours at room temperature and protected from light, then washed with PBS. Sections were mounted using Fluoromount-G mounting medium (SouthernBiotech) and then stored at -20°C for image acquisition. Images were captured using a Zeiss inverted laser scanning confocal microscope and Zen software. Z-stacks of pictures of the region of interest were generated for confocal imaging in increments of 1.0

m.

Stereotaxic Surgery

To manipulate expression of Fam107a, we used AAV1/2-CAG-FAM107A-IRES-eGFP-WPRE-bGHp(A) or AAV1/2-CMV-DIO-FAM107A_310122, or the respective control viruses (AAV1/2-CAG-IRES-eGFP-WPRE-bGHp(A), AAV1/2-CMV-DIO-eGFP). Animals are kept under Isoflurane (Floren, Abbott) inhalation-anaesthesia initializing at 4% Isoflurane in Carbogen (95% O_2, 5% CO_2) at a flowrate of 0,5 -1L/min, and lowering to 2% Isoflurane for the duration of the surgery. Mice were placed in a stereotaxic frame (Kopf Instruments) and body temperature was maintained with a heating pad. 30 minutes before surgery, mice were given s.c. injection of Metamizol at 200 mg/kg body weight and at the start of the surgery an s.c. injection of 0.5 mg/kg Meloxicam (Metacam®, Boehringer Ingelheim, Ingelheim am Rhein, Germany). AAV virus was bilaterally injected using a 33-gauge injection needle with a 5 µl micro-syringe (Hamilton®, Bonaduz, GR, Switzerland) utilizing an automated microinjection pump (World Precision Instruments) to inject 300 nl of virus at a rate of 100 nl/min. The targeted coordinates, relative to bregma, were as follows: -3.0 mm posterior, ±3.0 mm lateral, -3.2 mm ventral, which were confirmed post sacrifice. For the first 3 days after surgery, Metacam (5 mg/kg body weight) was mixed into the drinking water of the mice and administered as a systemic analgesic, and the body weight of the mice was monitored daily. After completion of the behavioral experiments, successful viral expression was verified by RNAScope.

Tissue collection and processing

For immunohistochemical analyses, mice were perfused with 4% (v/v) paraformaldehyde (PFA) under isoflurane anaesthesia, extracted brains preserved in 4% (v/v) PFA at 4°C for 24 hours and subsequently transferred to a 30% sucrose solution at 4°C until processing. For all other cohorts, animals were killed by decapitation under isoflurane anesthesia. The basal trunk blood was collected, the adrenal glands were removed and weighed, and brains were quickly frozen and stored at -80°C before being sectioned.

Golgi-Cox staining and analysis of dendrites and spines

Mice were anesthetized with isoflurane and decapitated, and immediately extracted brains were immersed in Golgi-Cox solution (8) for 14 days, followed by immersion in 30% sucrose solution for 5 days at room temperature in the dark. 120 µm thick serial coronal sections were cut on a Microm HM 650V vibratome (Thermo Scientific, Walldorf, Germany), mounted on

Superfrost plus slides (Thermo Scientific). Pyramidal neurons from the CA3 ventral hippocampus have been selected for structural analysis (6-8 neurons per area per animal). Neurons were traced at 40X and dendritic spines at 100X by using Neurolucida software (MicroBrightField Bioscience, Williston, VT). Sholl analysis was used to calculate the total dendrite length, and NeuroExplorer software (MicroBrightField) was used to count the connections at concentric circles (20 μm apart). Spine density was analyzed in segments of 10-40 μm in both apical and basal dendrites.

Supplemental Figures

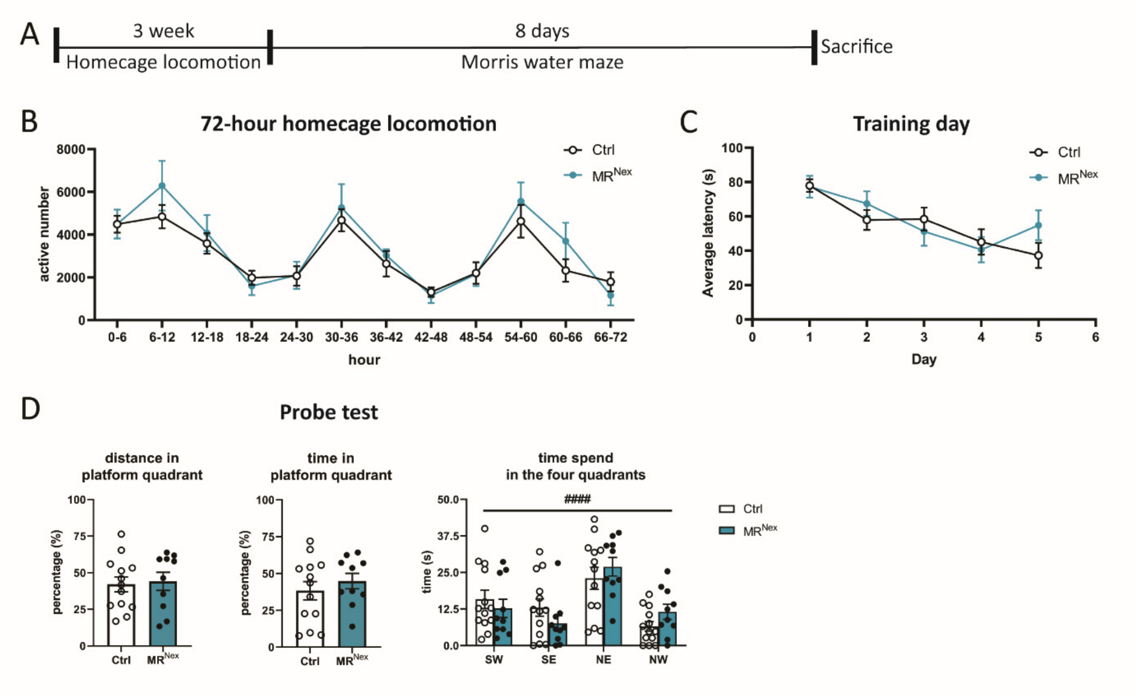

Figure S1 Male MR^{Nex} mice show no alterations in home cage locomotion and spatial learning. (A) Experimental design. **(B)** Both MR^{Nex} and Ctrl mice had obvious circadian rhythms in their home cage activity ($F_{(11, 108)}$=12.38, p<0.0001), but there was no significant difference between the two groups ($F_{(1, 108)}$=1.780, p=0.1849). N=8 for both groups. **(C)** Acquisition of spatial memory in the MWM was not different between the two genotypes **(D)** Loss of MR in glutamatergic neurons does not affect spatial learning in mice in the probe trial of the MWM ($F_{(1, 84)}$=0.002, p=0.963). MWM: Ctrl group: n=13, MR^{Nex} group: n=11. # indicates platform effect with $^{####}$ $p < 0.0001$.

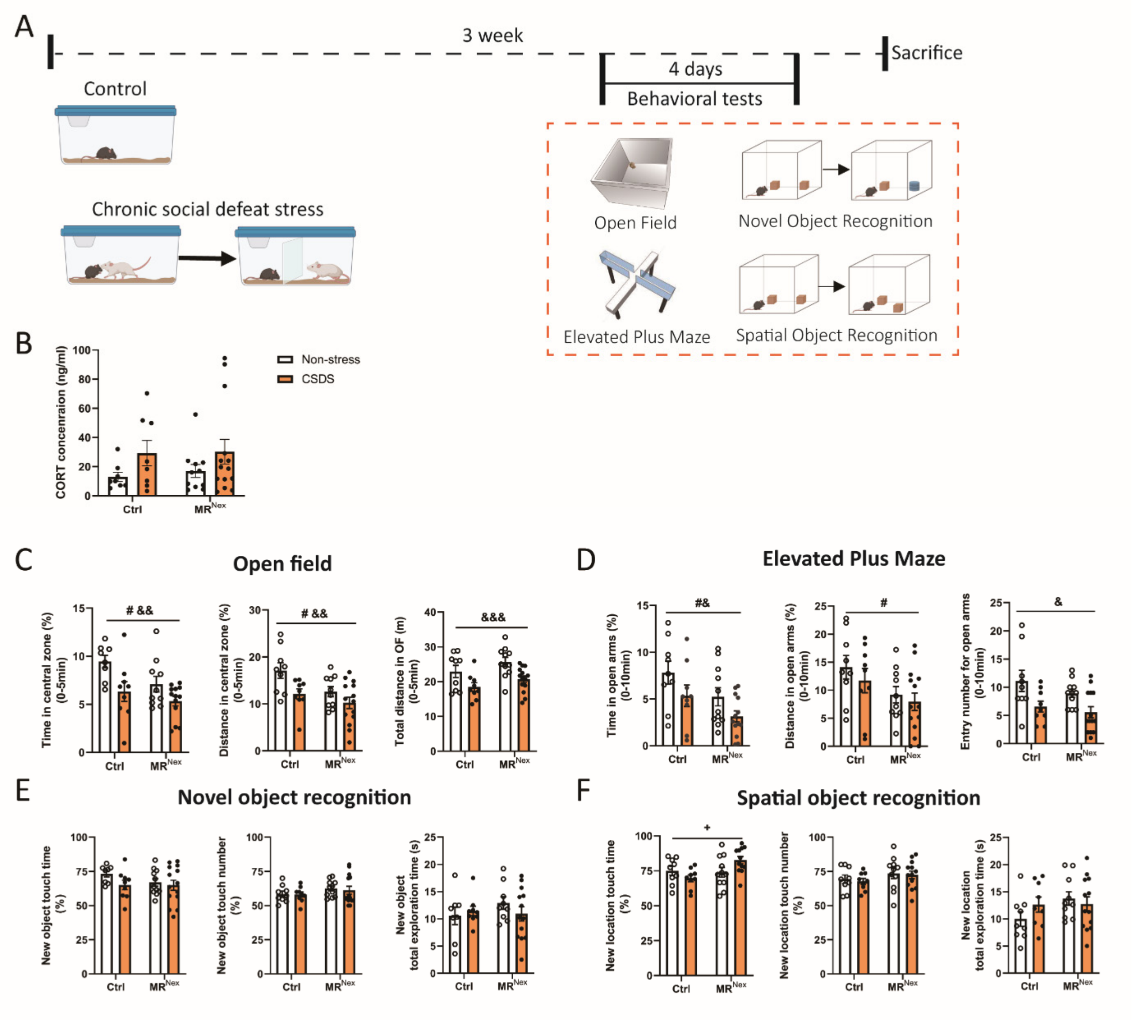

Figure S2 Chronic social defeat stress effects are independent of MR in glutamatergic forebrain neurons in male mice. (A) Experimental time line. **(B)** Basal CORT levels were elevated following CSDS (condition effect $F_{(1,37)}$=3.843, p=0.057), with no differences between genotypes ($F_{(1,37)}$=0.110, p= 0.742). In the OF test **(C)** central zone time and distance were significantly reduced by CSDS and genotype, with no interaction (center time: $F_{(1,36)}$=10.52, p=0.003; centre distance: $F_{(1,37)}$ =7.227, p=0.011). Total distance travelled was decreased by CSDS exposure ($F_{(1,39)}$=13.36, p=0.001). In the EPM test **(D)**, both time and distance in the open arms were decreased in MR^{Nex} mice, while time and entries in the open arms was decreased by CSDS exposure (open arm entries: ($F_{(1,38)}$=5.209, p=0.028); open arm time ($F_{(1,38)}$=5.663, p=0.022; open arm distance ($F_{(1,38)}$=5.598, p=0.023)), There was no genotype x CSDS interaction. In the NOR test **(E)** no changes related to stress exposure or genotype were detected (stress: percentage of time to explore the new object: $F_{(1, 38)}$=2.141, p=0.152; frequency of exploring new object: $F_{(1, 35)}$=0.174, p=0.679; total time spent exploring new object: $F_{(1, 39)}$=0.119, p=0.732; genotype: percentage of time to explore the new object: $F_{(1, 38)}$=0.867, p=0.358; frequency of exploring new object: $F_{(1, 35)}$=0.499, p=0.484; total time spent exploring new object: $F_{(1, 39)}$=2.180, p=0.148). No interaction of stress and genotype (percentage of time to explore the new position: $F_{(1, 38)}$=0.742, p=0.394; frequency of exploring new position: $F_{(1, 35)}$=1.22, p=0.277; total time spent exploring new position: $F_{(1, 39)}$=0.037, p=0.848). In the

SOR test **(F)**, there was a CSDS x genotype interaction, with MR^{Nex} mice showing an increased novel object touch time compared to controls following CSDS. while there was no obvious difference in the frequency of exploring the object in a new place (stress: $F_{(1, 37)}$=0.347, p=0.559; genotype: $F_{(1, 37)}$=2.010, p=0.165; interaction: $F_{(1, 37)}$=1.86, p=0.181) or the total amount of time spent exploring objects (stress: $F_{(1, 38)}$=0.198, p=0.659; genotype: $F_{(1, 38)}$=2.151, p=0.151; interaction: $F_{(1, 38)}$=0.009, p=0.9245), the percentage of MR^{Nex} mice touching the object in the new location was higher than that of Ctrl mice following CSDS. ANOVA detected a significant interaction of genotype and stress (stress: F(1, 37)=0.331, p=0.568; genotype: $F_{(1, 37)}$=3.953, p=0.054; interaction: $F_{(1,37)}$=5.590, p=0.023). Ctrl Non-stress: n=9, Ctrl CSDS: n=9, MR^{Nex} Non-stress: n=11, MR^{Nex} CSDS: n=13. # indicates genotype effect with $^{\#}p < 0.05$. & indicates CSDS effect with $^{\&}p < 0.05$, $^{\&\&}p < 0.01$, $^{\&\&\&}p < 0.001$. + indicates CSDS x genotype interaction effect with $^{+}p < 0.05$.

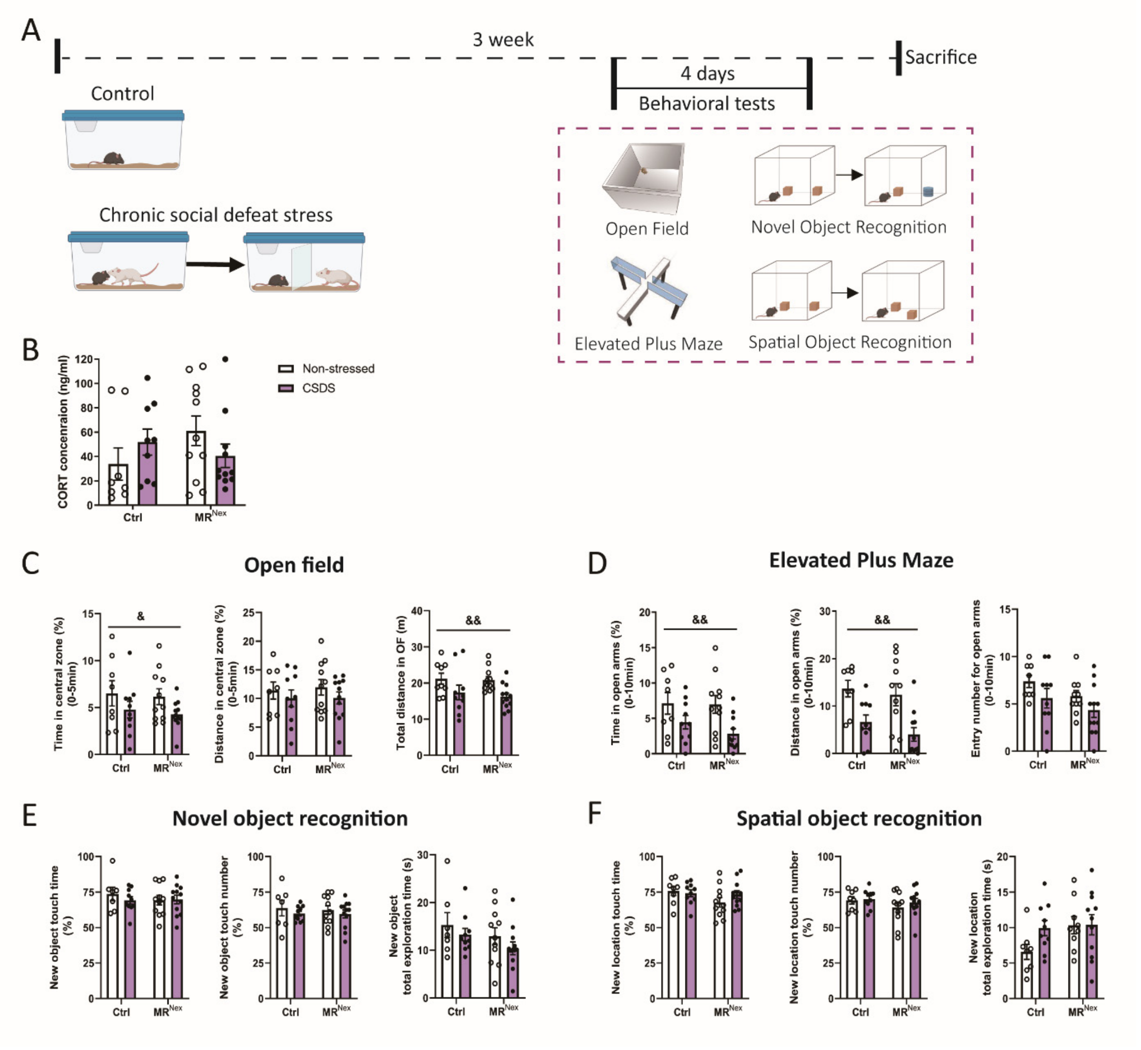

Figure S3 Chronic social defeat stress effects are independent of MR in glutamatergic forebrain neurons in female mice. (A) Experimental time line. **(B)** Basal CORT levels were not significantly affected by CSDS ($F_{(1,35)}$=0.013, p= 0.909) or genotype ($F_{(1,35)}$=0.485, p= 0.491). **(C)** In the OF, CSDS exposure significantly decreased time in the central zone ($F_{(1,37)}$=4.412, p=0.042) and total distance traveled ($F_{(1,37)}$=8.993, p=0.048), with no effect of MR genotype (percentage of time in central zone: $F_{(1, 37)}$=0.259, p=0.613; percentage of distance in central zone: $F_{(1, 37)}$=0.053, p=0.819; total distance: $F_{(1, 38)}$=0.313, p=0.579). **(D)** In the EPM, CSDS resulted in a significant decrease in open arm time ($F_{(1,36)}$=9.141, p=0.005) and distance ($F_{(1,36)}$=17.680, p<0.0001), with no genotype effects (percentage of time in open arms: F (1, 36)=0.628, p=0.433; percentage of distance in open arms: F(1, 36)=1.143, p=0.292; number of entries into open arms: F(1, 36)=3.088, p=0.087). In NOR test **(E)** and SOR test **(F)**, no significant differences were observed. In NOR test, the percentage of time to explore the new object did not affected by stress ($F_{(1, 36)}$=0.308, p=0.582) and genotype ($F_{(1, 36)}$=0.339, p=0.564); the frequency of exploring new object did not affected by stress ($F_{(1, 36)}$=2.290, p=0.139) and genotype ($F_{(1, 36)}$=1.742, p=0.195); total time spent exploring new object did not affected by stress ($F_{(1, 36)}$=0.058, p=0.811) and genotype ($F_{(1, 36)}$=1.017, p=0.320; Supplemental Figure 3E). In SOR test, the percentage of time to explore the new object did not affected by stress ($F_{(1, 37)}$=0.453, p=0.505) and genotype ($F_{(1, 37)}$=2.231, p=0.144); the frequency of

exploring new object did not affected by stress ($F_{(1, 34)}$=1.873, p=0.180) and genotype ($F_{(1, 34)}$=2.828, p=0.102); total time spent exploring new object did not affected by stress ($F_{(1, 37)}$=0.604, p=0.442) and genotype ($F_{(1, 37)}$=1.656, p=0.206). & indicates CSDS effect with $^{\&}p < 0.05$, $^{\&\&}p < 0.01$. Ctrl Non-stress: n=8, Ctrl CSDS: n=10, MR^{Nex} Non-stress: n=11, MR^{Nex} CSDS: n=12.

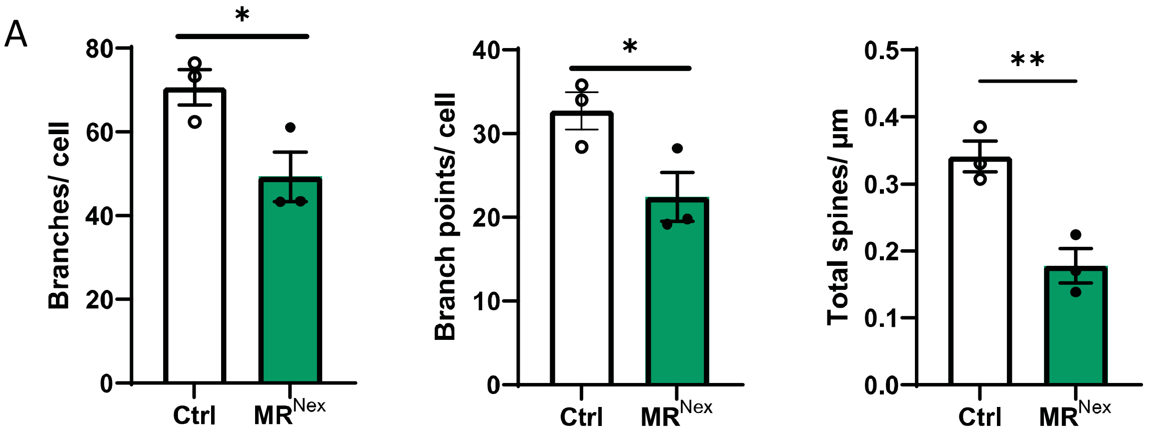

Figure S4 Deletion of MR in forebrain glutamatergic neurons leads to structural alterations. (A) MR deletion in glutamatergic neurons significantly reduced the dendritic branch number, branch points the number of spines in CA3 compared to Ctrl mice. Data obtained from n=3 mice per group, averaged per animal. *p < 0.05, **p < 0.01

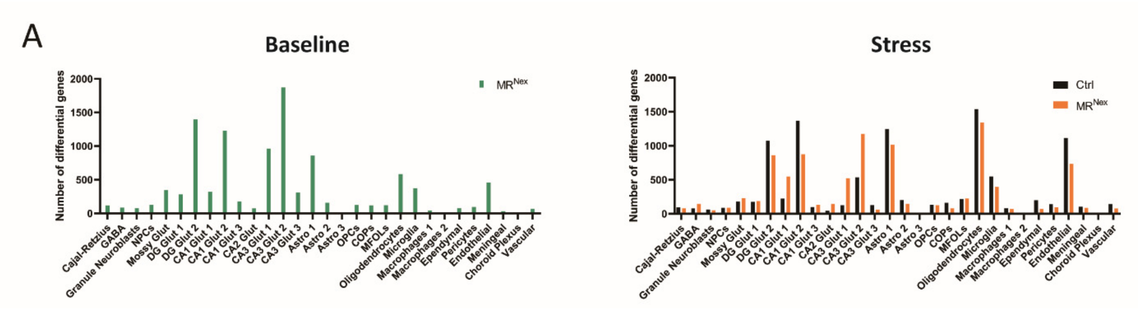

Figure S5 Single-cell RNA sequencing data of MR^{Nex} animals. (A) Histogram showing the number of differential genes in the baseline of MR^{Nex} animals (left panel) and following acute stress (right panel).

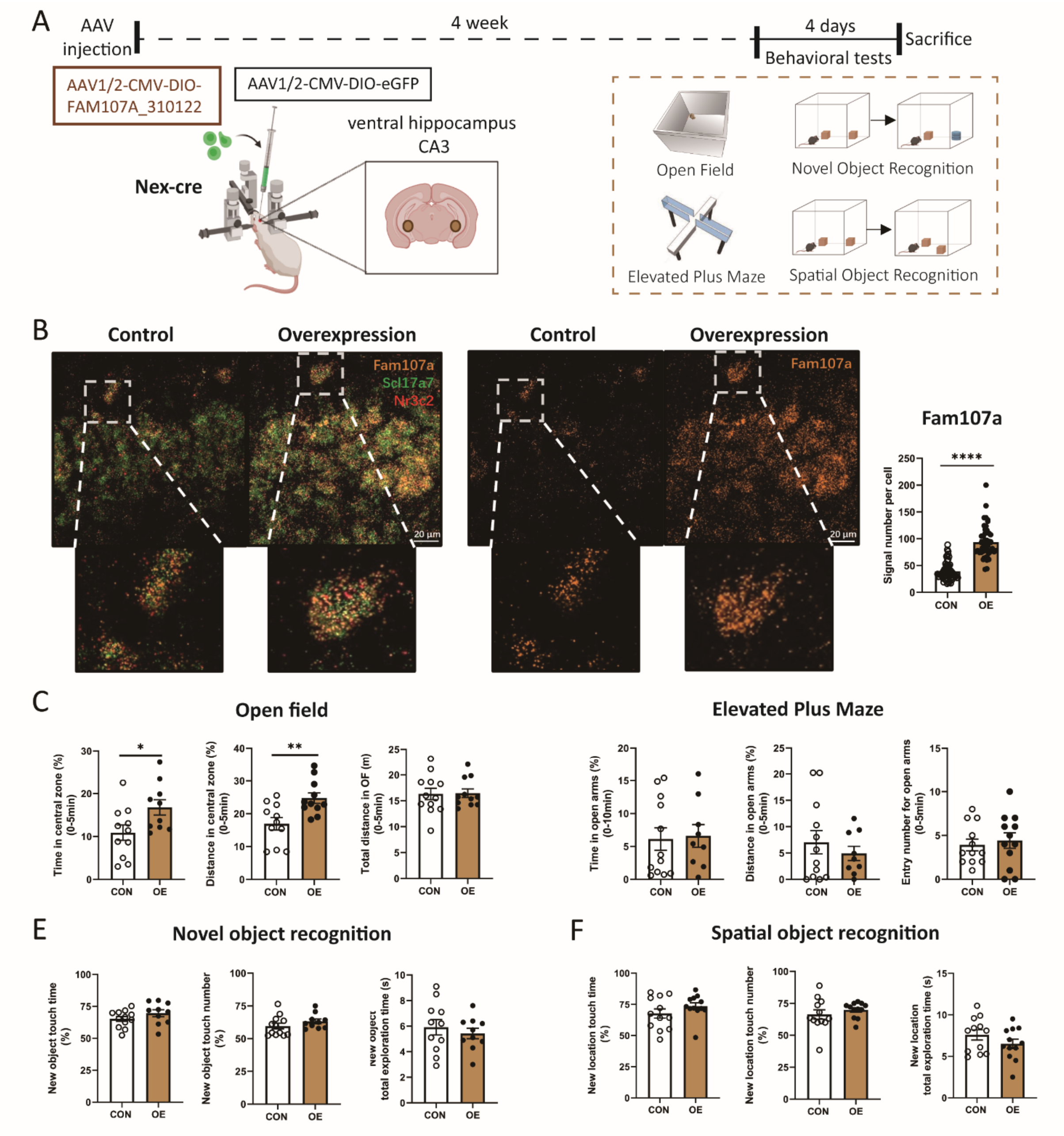

Figure S6 Fam107a overexpression in Nex-cre mice leads to reduced anxiety. (A) Experimental design. **(B)** The expression of Fam107a mRNA was significantly increased after virus injection ($t(105)=11.8$, $p<0.0001$). Left Panel: RNAScope images showing the mRNA expression of Fam107a (orange), MR (Nr3c2; red), and glutamatergic neuron-specific markers (Scl17a7; green). Right Panel: Individual channel RNAScope images showing the mRNA expression of Fam107a (orange). **(C)** In the OF test, Fam107a overexpression animals spent more time ($t(19)=2.336$, $p=0.030$) and covered more distance ($t(20)=3.180$, $p=0.005$) in the central zone. While there was no significant change in the overall distance travelled ($t(21)=0.104$, $p=0.918$). **(D)** In EPM, no significant behavioral differences were observed (percentage of time in open

arm: $t(19)=0.191$, $p=0.850$; percentage of distance in open arm: $t(19)=0.749$, $p=0.463$; open arm entry number: $t(22)=0.452$, $p=0.656$). In the NOR **(E)** and SOR **(F)** tests, there were no significant differences between the two groups. NOR: percentage of time to explore the new object ($t(20)=1.367$, $p=0.187$), frequency of exploring new object ($t(19)=0.646$, $p=0.526$), and total time spent exploring new object ($t(20)=1.224$, $p=0.235$). SOR: percentage of time to explore the new position ($t(22)=1.266$, $p=0.219$), frequency of exploring new position ($t(22)=0.893$, $p=0.382$), and total time spent exploring new position ($t(22)=1.293$, $p=0.209$). Con: n=12, OE: n=12. * indicates a significant difference with *$p < 0.05$, **$p < 0.01$.

References:

1. Berger S, Wolfer DP, Selbach O, Alter H, Erdmann G, Reichardt HM, *et al.* (2006): Loss of the limbic mineralocorticoid receptor impairs behavioral plasticity. *Proc Natl Acad Sci U S A* 103: 195–200.

2. Goebbels S, Bormuth I, Bode U, Hermanson O, Schwab MH, Nave K-A (2006): Genetic targeting of principal neurons in neocortex and hippocampus of NEX-Cre mice. *Genesis* 44: 611–621.

3. van Doeselaar L, Yang H, Bordes J, Brix L, Engelhardt C, Tang F, Schmidt MV (2021): Chronic social defeat stress in female mice leads to sex-specific behavioral and neuroendocrine effects. *Stress* 24: 168–180.

4. Mineur YS, Prasol DJ, Belzung C, Crusio WE (2003): Agonistic behavior and unpredictable chronic mild stress in mice. *BehavGenet* 33: 513–519.

5. Schmidt MV, Sterlemann V, Ganea K, Liebl C, Alam S, Harbich D, *et al.* (2007): Persistent neuroendocrine and behavioral effects of a novel, etiologically relevant mouse paradigm for chronic social stress during adolescence. *Psychoneuroendocrinology* 32: 417–429.

6. Sterlemann V, Ganea K, Liebl C, Harbich D, Alam S, Holsboer F, *et al.* (2008): Long-term behavioral and neuroendocrine alterations following chronic social stress in mice: implications for stress-related disorders. *Horm Behav* 53: 386–394.

7. Ennaceur A (2010): One-trial object recognition in rats and mice: methodological and theoretical issues. *Behav Brain Res* 215: 244–254.

8. Glaser EM, Van der Loos H (1981): Analysis of thick brain sections by obverse-reverse computer microscopy: application of a new, high clarity Golgi-Nissl stain. *J Neurosci Methods* 4: 117–125.
